# Infectious Endocarditis With Subarachnoid Hemorrhage and Cerebral Infarction in an Atopic Dermatitis Patient During Unsanitary Cupping Therapy

**DOI:** 10.7759/cureus.71599

**Published:** 2024-10-16

**Authors:** Mariko Okada, Yoshihiko Nakazato, Tokomo Yamaguchi, Eri Tominaga, Toshimasa Yamamoto

**Affiliations:** 1 Department of Neurology, Saitama Medical University, Saitama, JPN

**Keywords:** atopic dermatitis, cerebral infarction, cupping therapy, infectious endocarditis, subarachnoid hemorrhage

## Abstract

A 23-year-old man with a history of untreated atopic dermatitis (AD) presented with a headache and fever. The patient history revealed that he purchased a medical cupping kit on the internet and performed cupping therapy at the flexion of the right elbow joint without disinfecting. Brain computed tomography revealed subarachnoid hemorrhage in the right frontal sulcus, and brain magnetic resonance imaging indicated a small cerebral infarction. Cardiac ultrasonography revealed a mobile cord-shaped echo in the posterior apex of the mitral valve, which was later diagnosed as infectious endocarditis (IE). This case of IE in a patient with untreated AD was caused by a skin infection due to unsanitary cupping therapy.

## Introduction

Patients with severe atopic dermatitis (AD) are known to have a disorder of the skin barrier mechanism. As repeated skin curettage causes skin infections and bacteremia, preventing infectious endocarditis (IE) from occurring because of bacteremia is necessary [1]. Traditional Chinese medicine cupping therapy has been applied as an alternative therapy for various diseases, such as AD, in hospitals throughout China and worldwide [2]. Cupping therapy includes dry cupping and wet cupping. Of the two, wet cupping involves cutting the skin and placing a cup to aspirate blood. In Japan, cupping therapy is performed at medical institutions as an uninsured medical treatment. However, this treatment may have a high risk of skin infection if performed under unsanitary conditions. Herein, we report a case of a male patient in which a history of untreated AD caused the development of bacteremia, IE, and subarachnoid hemorrhage (SAH) owing to cupping therapy without disinfection.

## Case presentation

**Figure 1:**
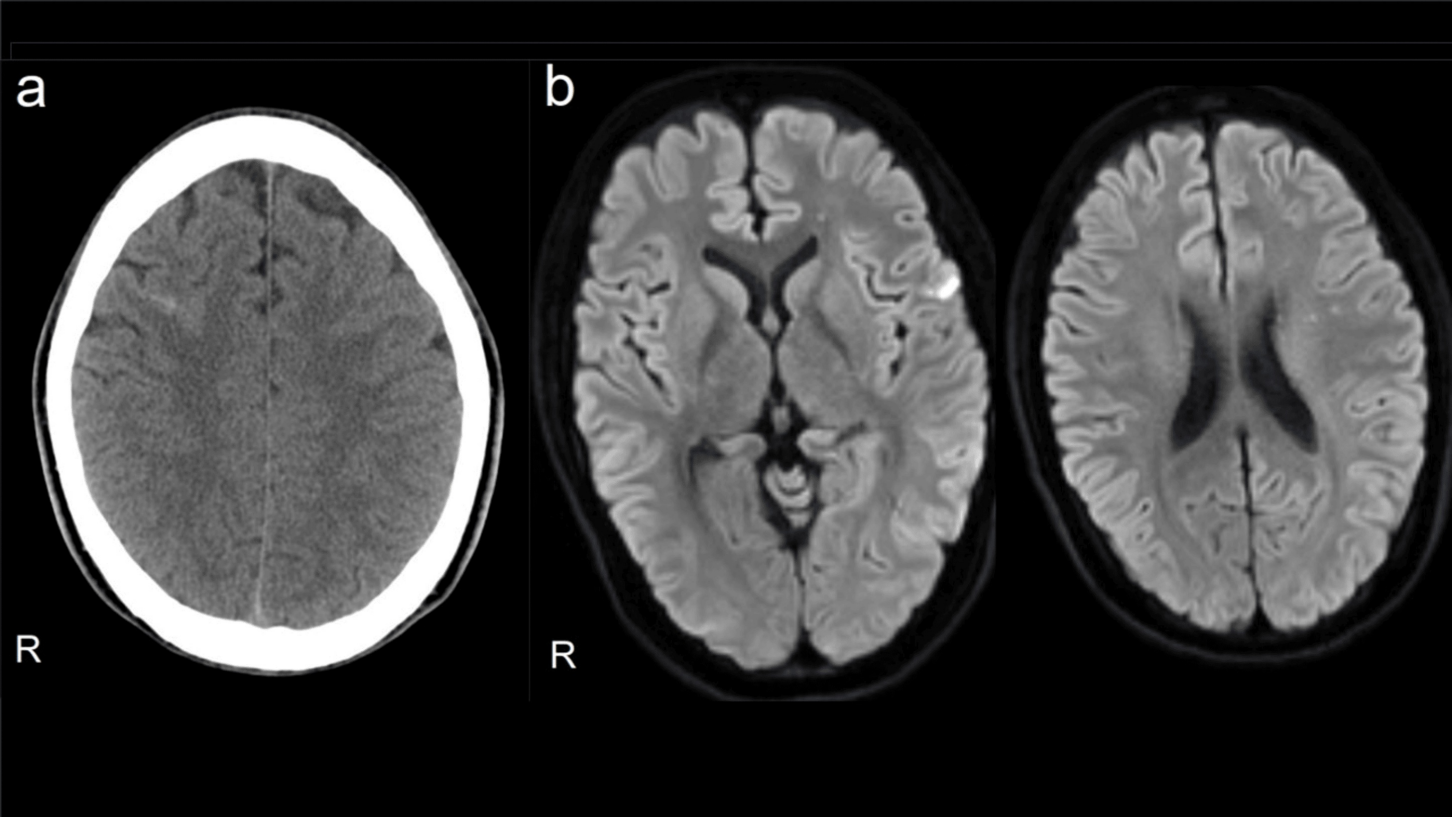
(a) Brain CT, showing SAH in the right frontal sulcus. (b) Brain MRI diffusion-weighted image, showing hyperintensity areas that appear in the left frontal lobe CT: computed tomography; SAH: subarachnoid hemorrhage; MRI: magnetic resonance imaging

A 23-year-old man who had been treated for AD in childhood had discontinued treatment on his own accord. Thirteen days before admission, he purchased a medical cupping kit on the Internet for the purpose of treating AD and performed it at the flexion of the right elbow joint without disinfection. Four days before admission, he developed a fever of 38°C and a throbbing headache. The next day, he noticed redness and swelling in his left index finger. The day before admission, the headache and fever did not subside, so he visited a home doctor, and on the same day, he was intravenously administered an antibiotic (ceftriaxone sodium 1 g/day) for three days. On the day of admission, his brain computed tomography (CT) scan revealed an SAH in the right frontal sulcus (Figure 1a), so he was transferred to our hospital.

His physical findings were as follows: body temperature, 38.1°C; pulse, 62 beats/minute; no arrhythmia; and blood pressure, 114/63 mmHg. Chest findings indicated normal respiratory sounds, no heart murmurs, swelling and redness at the tip of the left index finger, and mild purpura. The skin of the trunk and the limbs was dry, and mild AD was observed.

Neurological findings indicated clear consciousness and no neck stiffness, no abnormalities in cranial nerves, normal motor and sensory systems, and normal tendon reflexes. Blood tests revealed a slightly increased inflammatory response with a white blood cell count of 6,750/μL, hemoglobin count of 12.2 g/dL, platelet count of 13.9 × 104/μL, and C-reactive protein level of 1.95 mg/dL. Meanwhile, no abnormalities were observed in biochemical tests. Cerebrospinal fluid examination revealed a slightly increased cell count, with an initial pressure of 12.5 cmH_2_O, cell count of 7/μL (mononuclear leukocyte 5/μL and polymorphonuclear leukocyte 2/μL), protein of 25 mg/dL, and sugar of 51 mg/dL (blood glucose 96 mg/dL).

Hyperintensity areas were scattered in the left frontal cortex, and deep white matter of the left frontal lobe was detected in the brain magnetic resonance imaging (MRI) diffusion-weighted image at the time of hospitalization (Figure 1b). Even after hospitalization, the body temperature remained at 38°C to 41°C. Methicillin-sensitive *Staphylococcus aureus* (MSSA) was detected in two sets of blood cultures on the third day of hospitalization. Cardiac ultrasonography revealed a mobile cord-shaped echo with a diameter of 13 × 3 mm and a mild mitral regurgitation in the posterior apex of the mitral valve. Ejection fraction was maintained at 64%.

On the third day of hospitalization, decreased sensation in the left lower limb and hemiplegia of the right side occurred, and a new cerebral infarction lesion was detected in the MRI. Since the oscillating warts were observed on cardiac ultrasonography and MSSA was detected in the blood culture, IE was diagnosed according to the modified Duke diagnostic criteria [3]. The findings on the left index finger were considered Osler's node. On the fourth day of hospitalization, he was transferred to a cardiology center for mitral valvuloplasty.

**Figure 2:**
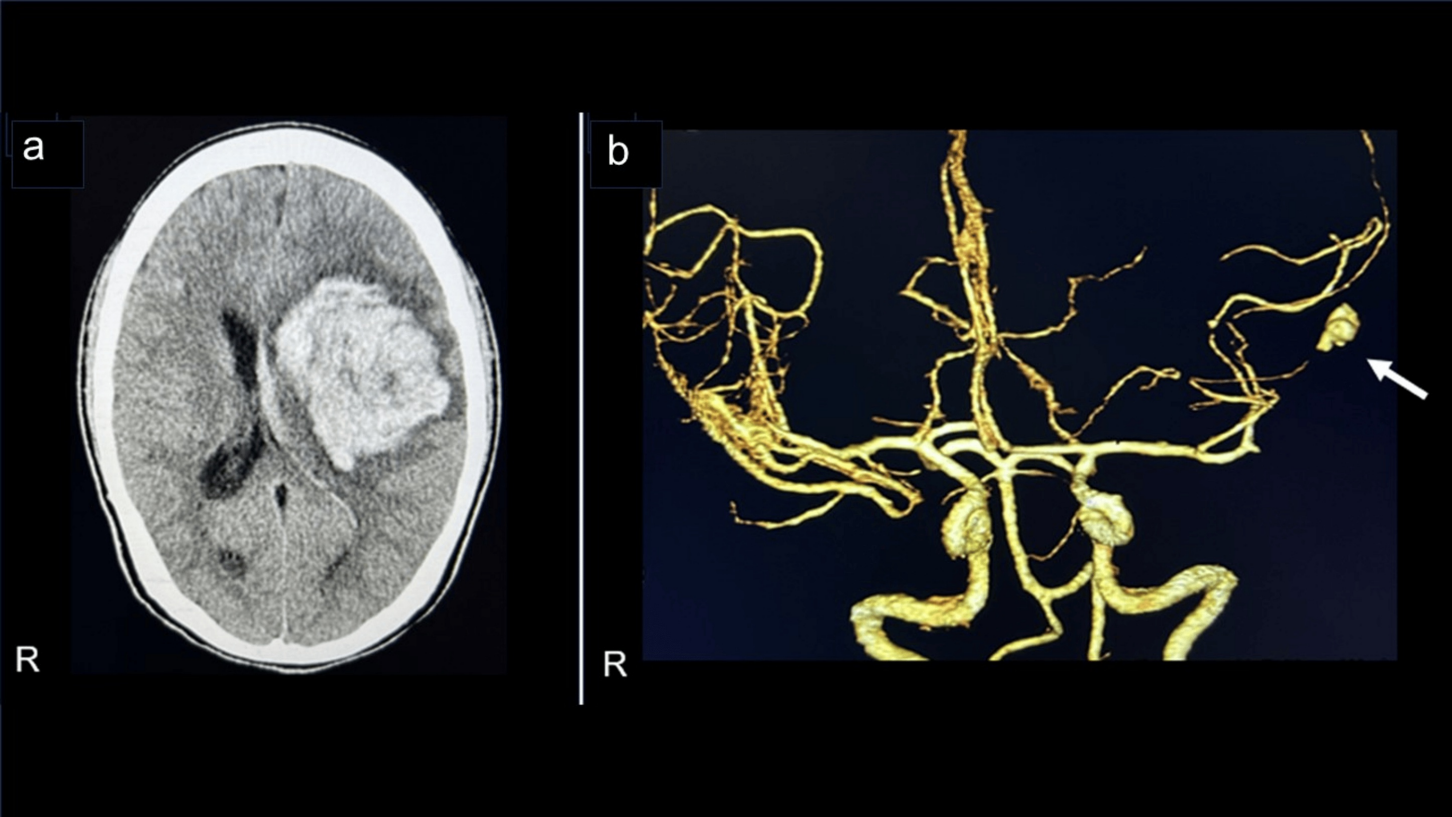
(a) Brain CT, showing intracranial hemorrhage in the left frontal lobe. (b) Cerebral angiography CT, showing aneurysm in the distal left middle cerebral artery (arrow) CT: computed tomography

In the early morning, three days after the transfer, he was observed to have impaired consciousness, generalized convulsions, and urinary incontinence. Since intracranial hemorrhage in the left frontal lobe and aneurysm in the distal left middle cerebral artery were observed by brain CT (Figure 2a) and cerebral CT angiography (Figure 2b), the intracerebral hemorrhage was considered to be caused by rupture of an infectious cerebral aneurysm, and craniotomy hematoma removal was performed on the same day. Mitral valvuloplasty was performed in the chronic phase after brain surgery.

## Discussion

In the present case, SAH developed following IE. The IE, in this case, was caused by a skin infection due to unsanitary cupping therapy with untreated AD. Severe AD often leads to dermatitis, bacteremia, and IE owing to the fragility of the skin barrier. In >90% of patients with AD, a large number of *S. aureus* was observed on the skin. Dermatitis was worsened by the patient's repeated curettage of the skin, and bacteremia was also occasionally present. Patients with AD who often develop IE due to bacteremia have been reported and have attracted attention [4]. The mean age of IE complications in AD was 28.4 years, and many patients did not treat AD for several years [1]. In this case, the patient was treated with topical steroids for AD in childhood; however, his AD had not been currently treated.

In addition, the incidence of central nervous system symptoms in IE is high at 60%-70% [5], and 47% of patients with IE are first affected by central nervous system symptoms [6]. In particular, cerebral infarction often develops in the early stages of IE onset, while SAH is rare [7,8]. In general, the risk factors for the development of IE include valvular heart disease, mitral valve prolapse, congenital heart disease, history of endocarditis, artificial valves, cardiac implantable devices, history of rheumatic fever, hemodialysis, and vascular catheterization [3]. IE was not initially suspected in this case because SAH was the first symptom, and these risk factors were not observed. However, after admission, multiple small infarctions involving the cerebral cortex on brain MRI and MSSA were detected in blood cultures, suggesting the diagnosis of cerebrovascular disease due to IE. Therefore, when the medical history was reexamined, it was discovered that wet cupping therapy was performed without disinfection. Thus, we considered that the IE was caused by the unsanitary cupping therapy performed on untreated AD. Cupping therapy has been reported to cause skin ulcers in patients with eczema, and caution has been issued regarding treatment indications [9].

The oscillation wart was confirmed by cardiac ultrasonography, MSSA was detected in the blood culture, and IE was definitively diagnosed with clinical symptoms. The risk factors for cerebral embolism in IE include the size of the tumor (≥10 or 15 mm, which increases after antibiotic administration), high mobility, the attachment site of the mitral valve (especially the anterior apex), and the causative bacteria Staphylococcus or fungi [3]. In this case, the risk of developing embolism was high, as the size of the wart, mobility, attachment to the mitral valve, and detection of MSSA were relevant. In addition, the formation of an infectious aneurysm caused SAH and intracerebral hemorrhage. The cause of IE, in this case, was considered to be skin infection due to unsanitary cupping therapy with untreated AD. He had no typical risk factors for IE.

## Conclusions

AD is prone to developing serious cerebrovascular disease caused by IE. Medical cupping kits can be purchased privately on the Internet, and this treatment seems to have a high risk of skin infection if the treatment is performed under unsanitary conditions. Self-use on medical cupping kits should be avoided in patients with AD.
